# The Prevalence and Incidence of Hemolytic Uremic Syndrome: A Systematic Review

**DOI:** 10.7759/cureus.39347

**Published:** 2023-05-22

**Authors:** Sarah S Aldharman, Shahad M Almutairi, Alaa A Alharbi, Meshal A Alyousef, Khalid H Alzankrany, Mohammed K Althagafi, Emtenan E Alshalahi, Khalid H Al-jabr, Abdullrahman Alghamdi, Syed F Jamil

**Affiliations:** 1 College of Medicine, King Saud Bin Abdulaziz University for Health Sciences, Riyadh, SAU; 2 Collage of Medicine, King Saud University, Riyadh, SAU; 3 Department of Psychiatry, King Salman Bin Abdulaziz Medical City, Medina, SAU; 4 College of Medicine, Imam Mohammad Ibn Saud Islamic University, Riyadh, SAU; 5 College of Medicine, Alrayan Medical College, Medina, SAU; 6 College of Medicine, King Abdulaziz University, Jeddah, SAU; 7 College of Medicine, Qassim University, Al-Qassim, SAU; 8 College of Medicine, Prince Sattam Bin Abdulaziz University, Al-Kharj, SAU; 9 Research, King Abdullah International Medical Research Center, Riyadh, SAU; 10 Pediatrics, King Abdullah Specialized Children's Hospital, Riyadh, SAU

**Keywords:** renal impairment, systematic review, incidence, prevalence, hemolytic uremic syndrome

## Abstract

A hemolytic uremic syndrome is an uncommon but severe condition brought on by an overactive alternative complement system, typically involving a hereditary component. It will be crucial to comprehend the epidemiology of hemolytic uremic syndrome as research advances toward bettering its diagnosis and treatment. A systematic review was conducted to evaluate the incidence and prevalence estimates of hemolytic uremic syndrome (HUS) internationally. A thorough literature search was conducted using PubMed, Springer, Cochrane Library for Systematic Reviews, and Embase databases between 2012 and 2023 in accordance with the Preferred Reporting Items for Systematic Reviews and Meta-Analyses (PRISMA) 2020 recommendations. A further source of data was the PubMed Central search engine. To make sure that the evaluation included just the studies that were the most pertinent, a population, interventions, comparators, and outcomes (PICO) eligibility criterion was also used. Eight articles were included in this review. HUS had an annual crude incidence of 0.66 per 100,000 people and a standard annual incidence of 0.57 per 100,000 people. Females were more likely than males to develop HUS, but only marginally more frequently. Patients under 20 years old were the age group where HUS was most common. HUS had an average cost of $21,500 per patient, which was more expensive than the country's overall inpatient average cost for the same period. This is due to patients requiring supportive care, antibiotics, plasma exchange, plasma infusion, and renal replacement therapy, and it could take multiple courses of treatment before they improve. It was concluded that several variables, including the region, the age group affected, and the frequency of the underlying bacterial infection, determine the prevalence and incidence of HUS. HUS is often more common in children than adults and is more common in some nations. Overall, HUS is an uncommon disorder that can have significant repercussions for people who have it. For better results and fewer consequences, HUS must be diagnosed and treated as soon as possible.

## Introduction and background

A hemolytic uremic syndrome (HUS) is a severe condition caused by an overactive alternative complement system and frequently includes a hereditary component [1]. Hemolytic anemia, thrombocytopenia, and renal impairment are the disease's main symptoms, impacting kidney function [2]. Complications outside the kidney that affect the central nervous, cardiovascular, pulmonary, gastrointestinal, and skeletal systems are frequent and can happen in up to 20% of patients [3]. Hemolytic uremic syndromes that fall into an unusual classification typically have causes other than cobalamin deficiency, streptococci, bacteria that produce the Shiga toxin, or other diseases [4,5]. Complement factor gene mutations, which are more common in youngsters, are thought to be responsible for the majority of HUS cases [6-8]. Males and females are equally affected by HUS in children, although females are more likely to get it in adult age [9]. Individuals with complement factor H mutations have less favorable prognoses and outcomes than those without genetic change [10]. 

When taking into account potential long-term health consequences that are not yet identified or assessed among cases, the possibility of underestimating the burden of HUS is increased. Consider how little attention has been given to the possible psychosocial effects of HUS on patients and their families. Given that HUS primarily affects young children - in 2017, the incidence of HUS among children under the age of five was more than double that for all pediatric cases, as previously cited - there may be severe psychosocial repercussions for family members who are responsible for caring for HUS survivors [11,12]. For instance, a recent study of 30 HUS case-parent dyads in Scotland found more significant anxiety about the future, changes in daily behavior, and emotional and psychological discomfort among the questioned parents [13]. Since there is no known cure for HUS, management of the condition is solely focused on supportive care, which includes copious fluid infusions, transfusions, anti-hypertensive medications, and renal replacement therapy when necessary [14]. According to studies, underhydrated patients perform worse than those who are normal or overhydrated in terms of neurological problems and renal survival [14,15]. In fact, a quick diagnosis can allow for a quick volume expansion that counteracts the harmful effect of hemoconcentration brought on by fluid loss through vomiting and diarrhea, which contributes to the progression of microvascular thrombosis and hypoxic/ischemic organ damage [16].

Global HUS disease epidemiology is not well known because the illness is uncommon [17]. When published, incidence and prevalence numbers are frequently coupled with other related conditions, such as thrombotic thrombocytopenic purpura or Shiga toxin-producing E. coli-linked hemolytic uremic syndrome, results in an estimation of the actual number of people with HUS that is incorrect [18]. Although some nations provide population-based figures, there are discrepancies in the incidence and prevalence of HUS worldwide. A thorough evaluation of the epidemiology is required to aid in furthering our understanding of the illness burden associated with HUS. Additionally, agencies that evaluate new technologies and health systems that provide them will need better estimates of HUS incidence and prevalence when better HUS diagnoses and treatments are created. To give a thorough perspective of the current epidemiological landscape, a study of HUS incidence and prevalence was conducted.

## Review

The research protocol and sources

The Preferred Reporting Items for Systematic Reviews and Meta-Analyses (PRISMA) 2020 guidelines were followed in conducting this systematic review [19]. Due to the vast array of clinical article databases that are accessible on the website, PubMed Central served as the primary data source. The medical databases Springer, Embase, Cochrane, and PubMed were used to perform thorough research on the prevalence and incidence of hemolytic uremic syndrome. Besides, since the systematic review only evaluated published literature, there were no ethical issues.

Literature search strategy

A detailed literature review demonstrating the prevalence and incidence of HUS was conducted using medical databases. The eligibility criteria are shown in Table 1. The computerized database search was guided by a list of predetermined keywords and, when appropriate, their associated synonyms. The accompanying data search string included field tags for search engine optimization, pertinent truncations, medical subject titles, and Boolean operators (and). The primary goal of the literature search was to find published, peer-reviewed resources on HUS. Search string: ("Hemolytic uremic syndrome OR "HUS'') AND ("incidence") AND ("prevalence") AND ("hemolytic anemia" OR ''renal impairment'').

**Table 1:** Eligibility criteria employed in this study HUS - hemolytic uremic syndrome

Inclusion criteria	Exclusion criteria
Strictly peer-reviewed research articles	Secondary information sources include systematic reviews, meta-analyses, reports, case series, and newspapers
Studies in English that have been accepted	Articles written in other languages
Primary studies were published between 2012 and 2023	Primary articles published prior to 2012
Studies exploring the incidence of HUS in human beings	Studies on the incidence of HUS in animals

Study selection and data extraction

Two different writers stratified the data acquired from the literature search. Duplicate and unnecessary data were eliminated using title and abstract screening. Then, each study that was discovered underwent a rigorous full-text examination. A Microsoft Excel (Microsoft® Corp., Redmond, US) spreadsheet was used to display the obtained data. Studies that either author felt should be included were provided on a separate sheet for the pooled analysis. Any discrepancies focused on the preliminary study, and after careful consideration, these discrepancies were settled through conversations. When there was disagreement, knowledgeable advice from respected experts was sought. The authors created a descriptive narrative table highlighting essential study elements for each study.

Results

Literature Search

The initial literature search uncovered 1196 publications on those aforementioned digital medical databases examining the prevalence and incidence of hemolytic uremic syndrome. After screening them, the systematic review took these studies into account. A further 211 studies were sourced from Springer, along with 116 from the Embase database, 167 from PubMed Central, 221 from the Cochrane Library, and 281 from the PubMed database. Seventy-seven studies were not included in the initial screening because they were duplicates. 

**Figure 1:**
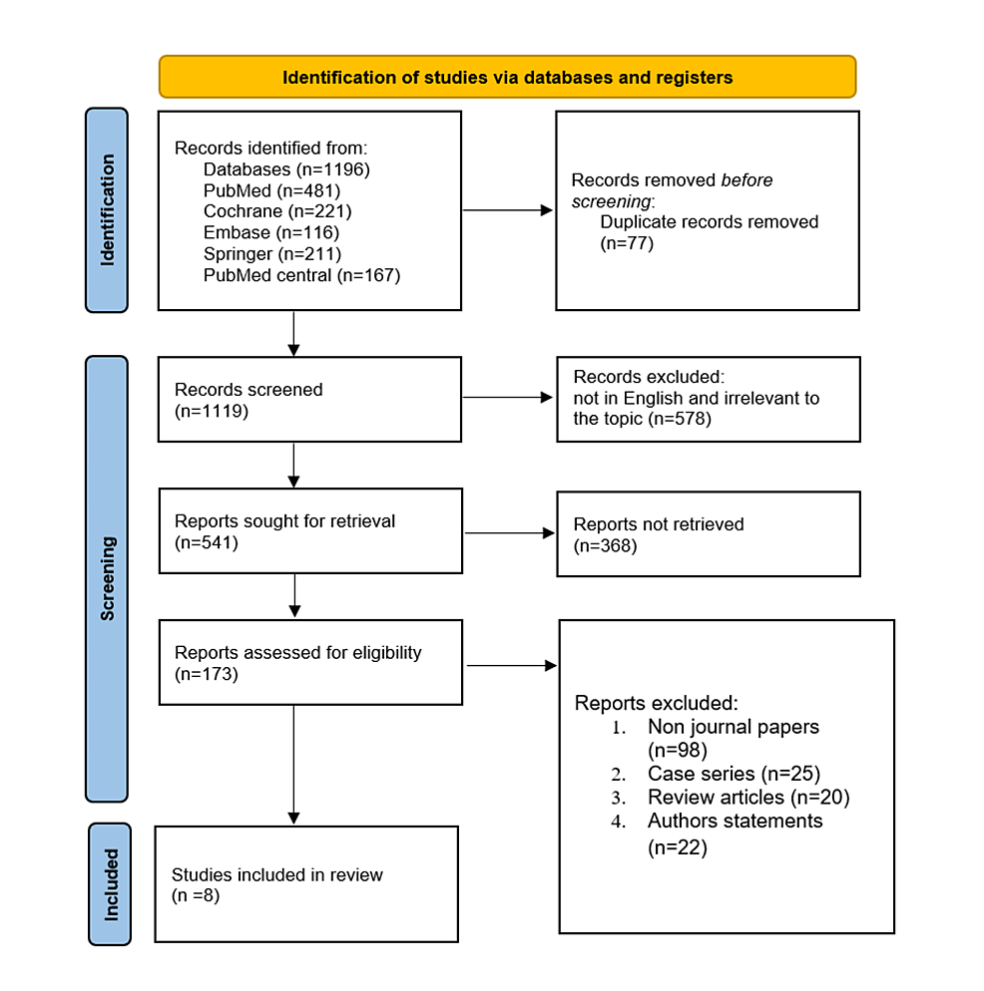
The flowchart showing the PRISMA strategy used to retrieve studies PRISMA - the Preferred Reporting Items for Systematic Reviews and Meta-Analyses

After two authors' initial titles and abstract screening on 1119 papers, 578 publications were excluded from the systematic review due to their non-English language and lack of relevance to the study's topic. As a result, 541 articles that satisfied the prerequisites underwent full-text analysis. Due to their failure to meet the requirements for inclusion, 368 studies could not be included in the review. The remaining 173 studies were further evaluated, including 20 reviews, 25 case series, 22 author comments, 98 critical and non-journal publications, methods, and 98 other publications. Eight were found suitable for inclusion in the study. The PRISMA flowchart shown in Figure 1 outlines the steps in conducting a literature search. Table 2 illustrates the characteristics of the included studies.

**Table 2:** Characteristics of the eight included studies in the review n/s - not specified

Study	Study design	Participants			Major outcomes (health outcomes)
		No	Age (mean, years)	Gender (female)	
Beczkiewicz et al. (2020) [20]	Cross-sectional study	74	n/s	44	Chronic disease, hypertension, severe stress, and acute sickness
Alfandary et al. (2020) [21]	Retrospective study	75	22	40	High blood pressure, bloody diarrhea, and fever
Ardissino et al. (2016) [22]	Observational study	101	n/s	46	Pneumonia, renal abnormalities, and methylmalonic acidemia
Capone et al. (2021) [23]	Retrospective study	100	n/s	39	Hemoglobinuria and bloody diarrhea
Ashida et al. (2018) [24]	Survey	258	n/s	140	Hypertension, diabetes, gastrointestinal disease, and cardiovascular disease
Robitaille et al. (2012) [25]	Retrospective cohort study	337	18	n/s	Renal complications, proteinuria, and hypertension
Alshaaili ​​​​​​​et al. (2018) [26]	Retrospective study	36	10.68	15	Abdominal pain, vomiting, hypertension, anemia, leukocytosis, and diarrhea
Jenssen et al. (2014) [27]	Retrospective study	47	n/s	32	Diarrhea

Quality Appraisal

Two different reviewers compiled the reliability and validity ratings of the studies selected for the systematic review. The studies' quality was evaluated using the Joanna Briggs Institute (JBI) assessment tool, a risk-of-bias assessment method that has received Cochrane approval [28]. 

Risk of Bias Evaluation

Based on the JBI evaluation tool, seven studies had moderate ratings, and one study had low ratings. Overall, the quality rating was moderate. Table 3 illustrate the quality assessment of included articles in this systematic review.

**Table 3:** JBI chart illustrating quality assessment of included articles in this systematic review Y - yes; N - no; L - low; M - medium; U - unclear; JBI - Joanna Briggs Institute

Checklist	Similar group and same population recruits	Exposure measurement	Valid/reliable measurement	Confounding factors identified	Confounding strategies	Groups free at the exposure	Outcomes measurement in valid/reliable way	Follow-up duration sufficiency	Completion of follow-up	Strategies to address incomplete follow-up	Appropriate statistical analysis	Quality rating
Author												
Beczkiewicz et al. (2020) [20]	Y	N	Y	Y	N	Y	Y	Y	Y	Y	Y	9/11 M
Alfandary et al. (2020) [21]	U	Y	Y	Y	Y	Y	Y	N	Y	Y	N	8/11 M
Ardissino et al. (2016) [22]	Y	Y	Y	N	Y	Y	Y	Y	Y	Y	Y	10/11 M
Capone et al. (2021) [23]	Y	Y	Y	Y	Y	Y	N	Y	Y	Y	N	9/11 M
Ashida et al. (2018) [24]	Y	U	Y	N	Y	N	Y	N	U	U	Y	5/11 L
Robitaille et al. (2012) [25]	Y	Y	Y	U	Y	Y	Y	Y	Y	Y	U	9/11 M
Alshaaili et al. (2018) [26]	U	Y	N	Y	Y	Y	Y	Y	Y	Y	Y	9/11 M
Jenssen et al. (2014) [27]	Y	Y	Y	Y	U	Y	N	Y	Y	N	Y	8/11 M

Discussion

HUS is a rare but severe condition that can cause acute kidney injury, anemia, and low platelet count. The prevalence and incidence of HUS can vary depending on several factors, such as age, geographical region, and underlying health conditions. According to a study published in the American Journal of Kidney Diseases in 2016, the incidence of HUS in the United States was estimated to be approximately 1.2 cases per 100,000 people per year [14,22]. The same study reported that the incidence was highest in children under the age of five, with a rate of 3.9 cases per 100,000 children per year [14].

In other countries, the incidence of HUS can be higher or lower. For example, a study conducted in France found an incidence rate of 0.62 cases per 100,000 people per year. In comparison, a study in Argentina reported an incidence rate of 7.9 cases per 100,000 children under the age of five [22-24]. The prevalence of HUS is difficult to estimate because it is a rare condition, and the exact number of people affected is not always known. However, the overall prevalence of HUS is estimated to be approximately two to three cases per 100,000 people. It's important to note that the incidence and prevalence of HUS can vary depending on the condition's underlying cause. For example, HUS can be caused by infection with certain strains of E. coli bacteria, which can be more prevalent in certain geographical regions or populations [20,25,27]. Other causes of HUS include certain medications, autoimmune disorders, and genetic mutations.

Since Shiga toxin-producing E. coli (STEC) infections are the most frequent cause of HUS and are frequently epidemic, there is a good chance that the incidence of HUS and STEC infections are connected. In several nations, in-depth research has been done on the epidemiologic characteristics of HUS. Incidences of HUS have been recorded in a wide range of numbers, depending on the populations studied and the diagnostic criteria used. Compared to the 2.1 cases per 100,000 person-years recorded in the United Kingdom and the 2.7 cases per 100,000 person-years in the United States, China had a lower incidence of HUS at 0.66 instances per 100,000 person-years [5,29]. HUS incidence rates have been reported to be lower than our findings in Australia (0.07 cases per 100,000 person-years) and Iran (0.28 cases per 100,000 person-years) [30]. The following variables might be to blame for these variations. First, we extrapolated data from the Urban Medical Insurance databases to determine our estimate of the incidence of HUS. According to certain studies, STEC-HUS may be more common in rural areas than in metropolitan areas [8,20-22]. Additionally, there is a decreased likelihood of gastroenteric infections with STEC due to better hygienic conditions in urban regions [31]. According to North American seroepidemiological surveys, rural people had higher antibodies against the O157 lipopolysaccharide rates than residents of urban areas [32]. Furthermore, HUS is a rare illness. It may be challenging for many doctors to diagnose HUS. Some hospitals in developing nations may lack the equipment and facilities needed for a precise diagnosis [8,26,33]. These elements might have led to an underestimation of the prevalence of HUS in most countries, for example, China. Third, it's still unknown how ethnic variables play a role. The genetic tendency to develop HUS may vary among the various studied populations [10,23,31].

In this study, females had a little greater incidence than males, although the difference was not statistically significant. Hypertension and cancer can both cause HUS [24,34,35]. Men have a greater crude incidence rate of cancer and a higher frequency of hypertension than women, which could be why men have a higher incidence of HUS [36]. Additionally, a greater incidence of HUS in women was found in numerous earlier research. The causes of the higher occurrence in female patients are unknown, but it may be partially explained by the fact that women are more likely to develop HUS after contracting E. coli O157:H7 gastroenteritis, and certain cases of HUS have been linked to pregnancy [37,38].

Children had the greatest incidence rate, with 5.08 per 100,000 person-years annually. But earlier studies from Europe and North America revealed that children under five were the most often impacted, and the age-specific incidence of HUS is comparable to that of STEC [20-22]. HUS incidence rates in children under the age of five were comparable to those in Australia but lower than those in Western Europe and the United States. This could be a result of the small percentage of patients with STEC-HUS in this study. To start with, persons who are exposed to STEC may have various habits and customs; for instance, Chinese people like completely cooked meat and boiled water. Second, compared to rural locations, urban areas have a lower rate of STEC infection [23,24,31]. The national average hospitalization cost per patient for the same time period was 1.30 thousand US dollars; however, the average hospitalization cost for each HUS patient was 1.75 thousand US dollars, which is much higher [25,26]. The overall cost per patient per year was 2.15 thousand US dollars, which was also ostensibly more expensive than the country's average for urban residents' medical care expenses [24,25]. In addition to supportive care, antibiotics, plasma exchange, plasma infusion, and renal replacement therapy are all used in the management of HUS [10,12,26]. The majority of these people must cover these expensive medical costs. For instance, a single plasma exchange or dialysis session costs around USD 15,000, and some patients require several sessions before fully recovering. 

Eculizumab has also been used successfully in patients with HUS in several nations, while it is still unavailable in others, such as China. The expense of this medication, which would amount to 350,000 US dollars a year for a child with a body weight of 30 kg, places a heavy financial burden on families [22,27,29]. After eculizumab is soon made available in China, medical expenses related to HUS might rise dramatically. Finally, it is important to note that certain publications have shown an increase in morbidity as a result of HUS. The prevalence of HUS in this new scenario becomes a critical signal for healthcare planning.

The prevalence of STEC infection is higher in rural areas; hence the annual incidence of STEC-HUS may have been overestimated. Second, because HUS is a rare condition, many doctors in underdeveloped nations could lack sufficient clinical knowledge about it. In hospitals without diagnostic tools, HUS incidence rates are likely underreported. Third, although it's possible that infants won't be insured for various reasons, most countries allow newborns to be covered by medical insurance for the first 90 days following delivery. Additionally, there was no information on the causes of HUS in patients. Further research is needed in order to improve preventative strategies and therapies.

## Conclusions

This systematic review summarizes the global incidence and prevalence estimates of HUS that are currently available. The prevalence numbers were found to be more variable, but overall, incidence estimates were comparable across all articles. Numerous epidemiological assessments on the frequency and incidence of HUS outside the majority of European countries are lacking, and there are various case definitions for the disease. This evaluation is crucial for healthcare providers all around the world because of the impact HUS has on patients and the healthcare insurance system. In addition, the research implies that the causes of HUS in nations like China may differ from those elsewhere, and doctors there may underestimate the prevalence of the disease because they don't fully comprehend it. From these angles, it is clear that additional etiological research, government assistance, and ongoing healthcare education are required in the future.
